# Exhaled Nitric Oxide and Pulmonary Oxygen Toxicity Susceptibility

**DOI:** 10.3390/metabo13080930

**Published:** 2023-08-08

**Authors:** David M. Fothergill, Jeffery W. Gertner

**Affiliations:** 1 Naval Submarine Medical Research Laboratory, Groton, CT 06349-5900, USA; 2 Naval Hospital Bremerton, Bremerton, WA 98312-1898, USA; jeffrey.w.gertner.mil@health.mil

**Keywords:** hyperoxia, pulmonary function, expired nitric oxide, spirometry, oxygen toxicity, diving, hyperbaric

## Abstract

Individual susceptibility to pulmonary oxygen toxicity (PO_2_tox) is highly variable and currently lacks a reliable biomarker for predicting pulmonary hyperoxic stress. As nitric oxide (NO) is involved in many respiratory system processes and functions, we aimed to determine if expired nitric oxide (F_E_NO) levels can provide an indication of PO_2_tox susceptibility in humans. Eight U.S. Navy-trained divers volunteered as subjects. The hyperoxic exposures consisted of six- and eight-hour hyperbaric chamber dives conducted on consecutive days in which subjects breathed 100% oxygen at 202.65 kPa. Subjects’ individual variability in pulmonary function and F_E_NO was measured twice daily over five days and compared with their post-dive values to assess susceptibility to PO_2_tox. Only subjects who showed no decrements in pulmonary function following the six-hour exposure conducted the eight-hour dive. F_E_NO decreased by 55% immediately following the six-hour oxygen exposure (*n* = 8, *p* < 0.0001) and by 63% following the eight-hour exposure (*n* = 4, *p* < 0.0001). Four subjects showed significant decreases in pulmonary function immediately following the six-hour exposure. These subjects had the lowest baseline F_E_NO, had the lowest post-dive F_E_NO, and had clinical symptoms of PO_2_tox. Individuals with low F_E_NO were the first to develop PO_2_tox symptoms and deficits in pulmonary function from the hyperoxic exposures. These data suggest that endogenous levels of NO in the lungs may protect against the development of PO_2_tox.

## 1. Introduction

Pulmonary oxygen toxicity (PO_2_tox) results from prolonged exposure to a hyperoxic atmosphere, with the severity of symptoms increasing progressively with elevation of the inspired oxygen partial pressure (PiO_2_) and the duration of exposure [1]. Symptoms of PO_2_tox include chest pain, tightness, cough, and substernal distress that may coincide with decreases in pulmonary function, specifically, a reduction in forced vital capacity (FVC) and alveolar diffusion capacity (D_L_CO) [1,2]. The toxic effects of oxygen are a concern for military and technical divers conducting prolonged multiday dives using oxygen rebreathers and for patients undergoing hyperbaric oxygen therapy or aggressive oxygen therapy for respiratory insufficiency at normobaric pressure. While there are theoretical models that predict the expected level of pulmonary function deficit because of prolonged exposure to raised PiO_2_ that are based upon the expected decline in FVC, there is considerable individual variation in susceptibility to a uniform degree of pulmonary oxygen poisoning [3,4,5]. Currently, there are no methods to predict individual susceptibility to PO_2_tox. Furthermore, a sensitive non-invasive biomarker that can detect changes in lung pathology at an early stage in the oxygen toxicity process has remained elusive.

Expired nitric oxide (F_E_NO) measurements have been studied as an exhaled marker of airway inflammation in a variety of lung diseases, including asthma, lung cancer, bacterial pneumonia, pulmonary fibrosis, and idiopathic pulmonary fibrosis [6,7]. Nitric oxide (NO) in expired air is derived from nitric oxide synthase (NOS) activity from various cellular sources, including neutrophils, alveolar type-II cells, endothelial cells, and airway cells, as well as from non-enzymatic sources such as s-nitrosothiols and nitrite protonation [6,8]. All three types of NOS (neuronal [nNOS], inducible [iNOS], and endothelium [eNOS]) have been identified in the human lung [9]. Endogenous NO in the lungs is thought to play an important role in host immune defenses by maintaining ciliary function, preventing the growth of bacteria and replication of viruses, modulating airway reactivity, facilitating surfactant production in the alveoli, and regulating inflammation and local blood flow in the lung [7,9].

The role of NO in the development or protection from O_2_ toxicity has been investigated in animal studies by several investigators [10,11] to better understand the roles of oxidative and nitrosative stress on hyperoxia-induced cell damage and acute lung injury [12,13]. Garat et al. [10] found that the survival time of hyperoxic rats treated with the NOS inhibitor NG-Nitroarginine Methyl Ester (L-NAME) was reduced compared to a hyperoxic control group, suggesting a protective effect of endogenous NO during 100% O_2_ breathing at normobaric pressure. Investigators at Duke University have shown that NO production may either exacerbate or mitigate the toxic effects of oxygen, depending on the NOS isoform that produces it [11,12]. These animal studies raise the intriguing possibility that individual variability in NO production in the lung may explain the large variability in individual susceptibility to PO_2_tox. Thus, the aim of this study was to determine if F_E_NO levels could provide an indication of PO_2_tox susceptibility in humans.

## 2. Materials and Methods

### 2.1. Subjects

The current study investigated individual differences to hyperbaric oxygen (HBO) stress using a small group of healthy, well-trained divers, rather than focusing on group mean changes in a larger, more diverse subject population. Consequently, the subject population was limited to qualified U.S. Navy-trained divers who were fit to dive and familiar with the signs and symptoms of both pulmonary and central nervous system (CNS) oxygen toxicity. Subjects were drawn from a convenience sample of U.S. Navy-trained divers (male or female) that were stationed at or worked at the Naval Submarine Medical Research Laboratory (NSMRL). During the informed consent process, all divers were reminded of the risks of pulmonary and CNS oxygen toxicity that could result from their participation in the study. Eight male U.S. Navy-trained divers aged 21–55 years (mean = 36.4 years), weighing 74.1–113.2 kg (mean = 91.8 kg), and with body stature ranging from 165–180 cm (mean = 174 cm) participated as subjects after signing the informed consent. The subject population consisted of enlisted divers and undersea medical officers, as well as civilian U.S. Navy-trained divers. All had normal lung function, were non-smokers, and abstained from all other diving activities for the duration of the study.

### 2.2. Study Design and Hyperbaric Oxygen Exposure Profile

The study protocol was approved by the NSMRL Institutional Review Board in compliance with all applicable federal regulations governing the protection of human subjects. The HBO exposures consisted of six-hour and eight-hour dry resting trials, breathing 100% humidified O_2_ at 202.65 kPa (2 ATA) in a hyperbaric chamber. The six- and eight-hour dives were performed on consecutive days. Pulmonary function and F_E_NO were measured immediately prior to each dive, between 10- and 60-min post-dive, and then daily for at least 3 days after the dive, until complete recovery of pulmonary function. Only subjects who showed no decrements in pulmonary function following the 6 h exposure conducted the 8 h dive. Based on previous studies, the level of pulmonary oxygen toxicity induced by 6 h of breathing 100% O_2_ at 2 ATA was predicted to cause a temporary decrease in FVC of between 4 and 6 percent in 50% of the subjects [2,3,4,14]. Extending the dive to 8 h increased the predicted decrement in FVC to 8% in 50% of the subjects [14]. Both dives were below the CNS oxygen toxicity limit (previous studies have shown no evidence of CNS oxygen toxicity in divers exposed to 2 ATA of oxygen for up to 12 h [3]). Due to the level of uncertainty in these predictions, it was felt that the current approach of conducting two dives with increasing length of oxygen exposure would permit measurable but fully reversible levels of pulmonary oxygen toxicity in our subject population, without exposing particularly susceptible individuals to an overly long HBO exposure.

During each dive, an inside tender who was not on oxygen accompanied the diver (subject). The hyperbaric oxygen exposure profiles were carefully selected to elicit a mild but reversible level of pulmonary oxygen toxicity in the majority of subjects while also keeping the risk of a seizure from CNS oxygen toxicity to a minimum. A single 15 min air break was incorporated during the midpoint of each HBO exposure, during which the subjects ate a low-nitrate/-nitrite lunch. The total bottom time of the dive was adjusted for the 15 min air break to ensure that the total time breathing 100% oxygen at 2 ATA was either 6 or 8 h. The initial six-hour HBO exposures were conducted in two teams of three and one team of two divers. All the dives were conducted at the same time of day (initial press between 07:10 and 08:36). Compression and decompression rates were 18.3 msw/min (60 fsw/min) and 9.1 msw/min (30 fsw/min), respectively.

Control exposures were conducted on two of the subjects to determine if pulmonary function or F_E_NO were significantly affected by breathing air at surface pressure in the hyperbaric chamber on the built-in breathing system for six or eight hours. One subject completed a six-hour air exposure, and the other subject completed an eight-hour air exposure. Both subjects completed the control exposures approximately four months after their oxygen exposures.

### 2.3. Pulmonary Function and Expired Nitric Oxide Measurements

Pulmonary function (FVC, forced inspiratory vital capacity [F_I_VC], forced expiratory volume in 1 s [F_E_V1]), the diffusion capacity for carbon monoxide (D_L_CO), and F_E_NO baseline measurements were collected from each diver twice per day (morning—am, and afternoon—pm) for five consecutive days before conducting the HBO exposures. During each measurement session, subjects conducted three repetitions for each pulmonary function test, meaning that the test met the American Thoracic Society (ATS) standards for repeatability [15,16,17,18]. All pulmonary function tests were conducted on the VMAX Encore 22 Pulmonary Function Module (Viasys Healthcare Inc., Yorba Linda, CA, USA). F_E_NO was measured using a chemiluminescence NO analyzer (Sievers NOA 280i, GE analytical instruments, Boulder, CO, USA). During each measurement session, F_E_NO was measured at the following 5 expired flow rates: 50, 100, 150, 200, and 250 mL/s. These were used to determine alveolar NO concentration (C_A_NO) and maximum airway wall flux of NO (J’awNO) using a two-compartment model [19]. Exhaled flow rates for on-line F_E_NO measurements were controlled by having the subject target the desired flow rate, presented on a computer screen, while expiring against a flow restrictor. Five different flow restrictors were used to achieve the different expired flow rates. At each expired flow rate, the mean value from at least three F_E_NO measurements that conformed to the standardized procedures recommended by the American Thoracic Society for online F_E_NO measurement [20] were taken during each measurement session and used in the analysis. The Sievers NO analyzer and VMAX Encore 22 Pulmonary Function Module were calibrated in accordance with the manufacturer’s procedures at least twice daily (morning and afternoon) before each measurement session. During each measurement session, the subjects conducted the F_E_NO measurements before the pulmonary function tests to avoid the potential influence of the spirometry measurements on F_E_NO. Pre-dive measurements for NO and pulmonary function were taken during the two-hour period before the dive. Post-dive measurements of F_E_NO were initiated 10 min after the dive had reached the surface. As D_L_CO measurements were always conducted after the F_E_NO measurements, subjects breathed ambient air for at least 20 to 30 min following the dives before conducting their first D_L_CO measurement. Consequently, P_A_O_2_ levels were expected to be at normal levels during the pulmonary function test and, thus, no corrections were made to D_L_CO for P_A_O_2_.

### 2.4. Data Analysis

A decrement in pulmonary function for an individual was defined as outside their normal variability if one or more of their pulmonary function tests (i.e., FVC, F_I_VC, F_E_V1, or D_L_CO) fell more than two standard deviations (SD) below their mean baseline value for that test. A change in F_E_NO was also considered outside normal variability if the change was greater than 2 SDs from the individual’s mean baseline F_E_NO value. Intra-individual variability for F_E_NO and the various pulmonary function tests were expressed as 2 × the coefficient of variation (CV), where CV = (SD/mean) × 100, to facilitate the comparison among individuals and between variables with different units and different means. All statistical analyses were carried out using Statistica software (Statsoft Inc., Tulsa, OK, USA). Analysis on the effect of time of day (am or pm) on F_E_NO across all expired flow rates was performed using a repeated measures analysis of variance (ANOVA). Repeated measures ANOVA was also used to compare group mean changes in F_E_NO, C_A_NO, J’awNO_,_ and pulmonary function at the different time points. When significant main effects of time were observed, the Dunnett post hoc test was used to explore differences between baseline and post-dive values. The relationships between F_E_NO levels and the percent changes in pulmonary function following the six-hour dive were evaluated using linear regression and the Pearson Product moment correlation coefficient. Significance was set at *p* < 0.05.

## 3. Results

Baseline individual means, 2 × the coefficient of variation (2 × SD/mean × 100%) for F_E_NO (at 50 mL/s expired flow rate), and the different pulmonary function tests (i.e., FVC, F_I_VC, D_L_CO) that were derived from the twice daily measurements (am and pm) taken on five consecutive days (*n* = 20 data points per mean per subject for each pulmonary function test) are shown in the second column of Table 1, Table 2, Table 3 and Table 4. The remaining columns in each table show the percent change in that variable from each individual’s mean baseline level following the HBO exposures. In each of the tables, the subject’s data are ordered from highest (top) to lowest (bottom) baseline F_E_NO. Additional tables showing changes in D_L_CO adjusted for Hb (D_L_CO_adj_), D_L_CO adjusted for alveolar volume (D_L_CO/VA), alveolar volume (V_A_), and F_E_V1 are presented in Appendix A (Table A1, Table A2, Table A3 and Table A4).

As shown in Table 1, there was a threefold range (19 to 59 ppb) in the baseline F_E_NO between subjects. Analysis of the F_E_NO baseline data using all expired flow rates indicated a significant time of day effect, with F_E_NO on average 10% lower in the afternoon compared to morning (*p* < 0.001). There was, however, no difference detected between the pre-dive F_E_NO taken on the morning before the six-hour dive and the baseline F_E_NO (*p* = 0.9994). Immediately following the six-hour oxygen exposure, all eight subjects had significant decreases in F_E_NO (i.e., values > 2 × CV less than their baseline), with the group mean change showing a 55% decrease (*p* < 0.0001). By the morning after the dive, F_E_NO levels had returned to normal in the majority of divers (six out of eight).

The four subjects with the lowest baseline F_E_NO and lowest post-dive F_E_NO (subjects 2, 3, 4, and 9) had clinical symptoms of pulmonary O_2_ toxicity and showed significant decreases in pulmonary function on one or more of the pulmonary function tests immediately following the six-hour exposure (see Table 2, Table 3 and Table 4). The clinical symptoms reported included chest fullness/tightness, congestion, mild substernal burning, and tickling or cough on deep inhalation. Subjects 1, 5, 7, and 8, who had baseline F_E_NO levels greater than the group mean of 34 ppb, showed no pulmonary function deficits or symptoms of pulmonary O_2_ toxicity following the six-hour HBO exposure and, thus, conducted the eight-hour HBO exposure the following day. Immediately following the eight-hour dive, three of these subjects had pulmonary function deficits (see Table 2, Table 3 and Table 4) and all four subjects showed greater decreases in F_E_NO than following their six-hour dive (mean ± SD F_E_NO post-dive 1 vs. post-dive 2 = 22.2 ± 3.4 ppb vs. 16.6 ± 2.7 ppb, respectively, *n* = 4, *p* < 0.01). Subject 5 had the highest baseline F_E_NO and was the only subject who did not show symptoms of PO_2_tox or a pulmonary function deficit following the HBO exposures.

During the three days following the dives, five subjects showed significant increases in F_E_NO (see Table 1). However, the timing of these increases and the duration of the elevated F_E_NO was variable among the subjects. Consequently, the group analysis did not reveal any statistically significant change in the mean F_E_NO from the pre-dive baseline during recovery days one (*p* = 0.8642), two (*p* = 0.0579), or three (*p* = 0.3358).

The pulmonary function test that demonstrated the greatest number of significant decrements following the oxygen exposures was D_L_CO (see Table 4). The three subjects with the lowest baseline F_E_NO (subjects 4, 3, and 9) had the greatest relative decrements in D_L_CO, which persisted for one to three days post-exposure. Subjects 1 and 8 also showed significant decreases in D_L_CO during the recovery period. When D_L_CO was corrected for V_A_, all subjects except subject 5 showed significant decrements at some point during the recovery period (see Table A2 in Appendix A). Both D_L_CO and D_L_CO/VA showed a significant main effect of time (*p* < 0.05 and *p* < 0.01, respectively) that was predominantly due to lower values during the second day of recovery compared to baseline (see Table 4 and Table A2).

**Figure 1:**
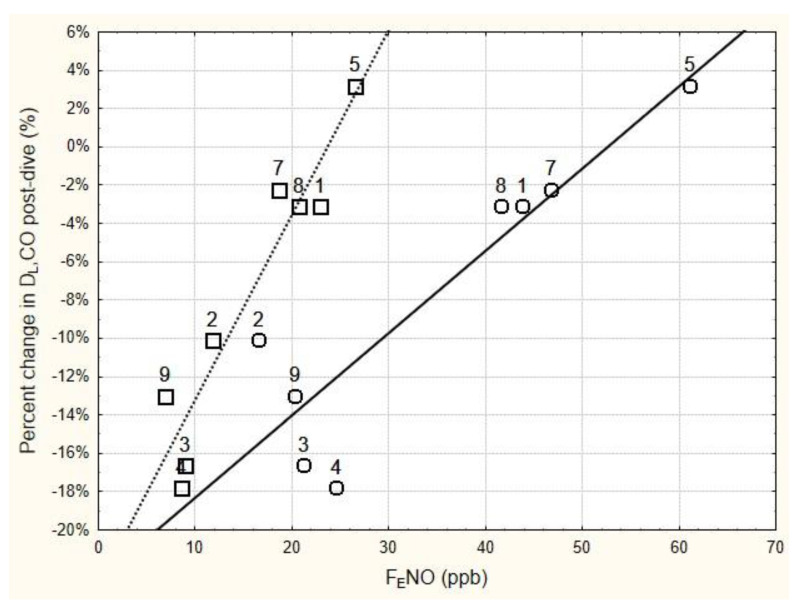
Relationship between the change in D_L_CO immediately following 6 h of breathing 100% oxygen at 202.65 kPa, and the immediate pre-dive F_E_NO (circles) and post-dive F_E_NO (squares). F_E_NO was measured at 50 mL/s expired flow rate. The numbers next to the data points are subject number identifiers. Subjects 3, 4, and 9 all showed significant decrements in D_L_CO immediately post-dive (see Table 4). Regression equations for the solid line and dashed line are as follows: Percent change in D_L_CO = −0.2265 + 0.0043 × pre-dive F_E_NO (r = 0.9018, *p* = 0.0022; r^2^ = 0.8132); Percent change in D_L_CO = −0.2286 + 0.0096 × post-dive F_E_NO (r = 0.9485, *p* = 0.0003; r^2^ = 0.8996).

The relationship between the relative change in D_L_CO immediately following the six-hour dive and the immediate pre- and post-dive levels of F_E_NO is shown in Figure 1. Regression analysis of these data found that the relative change in D_L_CO immediately post-dive was significantly related to the immediate post-dive F_E_NO (r = 0.948, *p* < 0.001), as well as to the pre-dive F_E_NO (r = 0.902, *p* < 0.01). Using the mean baseline F_E_NO in the regression analysis instead of the pre-dive F_E_NO slightly improved the relationship (r = 0.931, *p* < 0.001).

While some subjects had significant decrements in the spirometry tests following the dives, the group mean relative changes in the spirometry tests were on average smaller than those found for D_L_CO. Immediately following the six-hour dive, F_I_VC appeared to be more affected than FVC; however, neither F_I_VC nor FVC showed a significant main effect of time following group analysis (*p* = 0.0658 and *p* = 0.2176, respectively).

The two-compartment model analysis of the F_E_NO data showed a significant 58% decrease in J’awNO (mean ± SD, baseline vs. post-dive 1 = 1681 ± 747 pl/s vs. 709 ± 465 pl/s, *p* < 0.001), with no change in C_A_NO (mean ± SD baseline vs. post-dive 1 = 2.9 ± 1.2 ppb vs. 2.6 ± 0.6 ppb; *p* = 0.995) immediately following dive 1. A comparison of pre- and post-exposure measurements for F_E_NO and pulmonary function for the two subjects who conducted the surface control trials showed that all the dependent variables following the control exposure were within each individual’s normal daily variability.

## 4. Discussion

Traditionally, the “gold standard” for assessing PO_2_tox has been to measure changes in pulmonary function using spirometry (i.e., FVC) or D_L_CO. However, the sensitivity of these pulmonary function tests to assess PO_2_tox susceptibility has been questioned [21,22], and more recent research has explored other components in exhaled breath as potential biomarkers of PO_2_tox [22,23,24,25,26,27]. Since the initial discovery that NO was present in expired air [28], F_E_NO has been one of the most widely studied exhaled breath biomarkers of pulmonary health. F_E_NO increases significantly in a variety of inflammatory airway diseases and is now commonly used to diagnose and phenotype asthmatics [7]. It was thus originally hypothesized that F_E_NO would increase following HBO exposure due to free oxygen radical initiation of inflammatory reactions in the lungs.

One of the first published papers on the effect of hyperoxia on F_E_NO reported that F_E_NO increased with exposure to normobaric hyperoxic gas mixtures [29]. However, the 10 min normobaric oxygen exposures in this early study were unlikely to result in inflammation of the lungs. The results of the study by Schmetterer et al. [29] are in direct contrast with our finding of a marked acute reduction in F_E_NO following prolonged HBO exposures. One potential reason for the disparate results is that, at the time that Schmetterer et al. [29] performed their study, there was no standard method for measuring F_E_NO. Since that time, it has become clear that F_E_NO is highly dependent on the expired flow rate and, thus, the recommended guidelines for F_E_NO measurements published by the ATS in 2005 [20] have since standardized expired flow rates at 50 mL/s using a flow resistor that also prevents contamination of the F_E_NO measurement from the high levels of NO found in the nasal cavity. Although we did observe significant increases in F_E_NO during the recovery days in five subjects, which may be reflective of a delayed inflammatory reaction in the lungs, only one of these subjects (subject 9) exhibited consistent decrements in pulmonary function during all three recovery days that was concomitant with abnormally elevated F_E_NO levels.

A second main finding from our study is that the duration of the HBO exposure affected the relative magnitude of the post-dive decrease in F_E_NO, with the eight-hour HBO dive resulting in significantly lower post-dive F_E_NO levels than the six-hour HBO exposure. This finding implies that the magnitude of the temporary F_E_NO decrease following the HBO exposures may be dose dependent. Since conducting these pilot HBO dives in 2007, we have conducted a wide variety of dry human hyperoxic exposures with varying inspired oxygen partial pressures and exposure durations to determine if the F_E_NO decreases found in the current study follow a predictable dose–response relationship. Findings from these studies have been presented to the undersea and hyperbaric medical and research community at various scientific forums [30,31,32] and were summarized in preliminary form by Fothergill and Weathersby [33]. This study showed that the relative change in F_E_NO following dry resting hyperoxic exposure follows an exponential decline that is tightly related to the hyperoxic dose of the preceding exposure [33]. In the statistical model of the changes in F_E_NO with varying HBO exposures, Fothergill and Weathersby [33] used the following expression to define the hyperoxic dose of the HBO dives based upon the inspired partial pressure of the oxygen breathed (PiO_2_) and the duration of the exposure:

Hyperoxic Dose (ATA.min) = [PiO_2_ (ATA) × Exposure Duration (min)] − [0.21 × Exposure Duration (min)] (1)

Other investigators have also reported acute decreases in F_E_NO levels following HBO exposures [34,35,36,37,38,39,40]; however, the oxygen dose involved in these studies has rarely been great enough to induce changes in lung function or PO_2_tox symptoms noticeable enough to determine if the F_E_NO changes were related to PO_2_tox susceptibility. Our study is, therefore, somewhat unique, in that we were able to observe symptoms of PO_2_tox and measure significant decreases in lung function in some of our subjects, and then relate them to the observed changes in F_E_NO. Based upon our observations, we found that those individuals who had the lowest pre-dive F_E_NO levels exhibited the lowest post-dive F_E_NO levels and were most susceptible to PO_2_tox.

This significant linear relationship between the pre-dive baseline levels of F_E_NO and the relative decrease in D_L_CO measured immediately post-dive should be taken with caution when interpreting the effects of HBO exposure on PO_2_tox susceptibility. In a more recent study, in which healthy U.S. Navy-trained divers were exposed to 6.5 h of 100% O_2_ at 2.0 ATA [27], one subject, who aborted the dive early due to severe PO_2_tox symptoms, was found to have a 15% increase in D_L_CO immediately post-dive compared to his pre-dive base line [41]. Concomitant with the increase in D_L_CO was a 125% increase in total airway resistance and a 35% increase in proximal airway resistance (as measured using an impulse oscillometry methodology) [41]. We surmise that the elevated D_L_CO post-dive for this subject was an artifact caused by the increase in pulmonary resistance that resulted in a large negative interpulmonary pressure being generated during the fast inspiratory maneuver required to perform the D_L_CO measurement. The negative interpulmonary pressure could result in increased blood volume entering the lung before the D_L_CO breath-hold maneuver, raising the potential sink for the inhaled carbon monoxide gas mixture and artifactually raising the D_L_CO level. Therefore, we hypothesize that subjects who are particularly susceptible to PO_2_tox might experience a narrowing of the airways, possibly due to loss of normal airway tone.

Acute changes in airway diameter can be evoked by increases in cholinergic nerve activity or withdrawal of nitrergic neural activity [42]. Interestingly, noncholinergic neurotransmitters such as NO are thought to control human airway smooth muscle and normal airway tone via nitrergic parasympathetic nerves [42]. Thus, factors that compromise normal nitrergic parasympathetic control of airway tone, such as reduced levels of NO, would cause narrowing of the airways. The acute post-dive increase in airway resistance seen in the above PO_2_tox case was concomitant with an extremely low post-dive F_E_NO of 3.5 ppb [41]. This is consistent with a neurogenic PO_2_tox response rather than an inflammatory reaction to the HBO exposure.

Although our study was not designed to elucidate the underlying mechanisms responsible for the reduction in F_E_NO with HBO exposure, our results are consistent with the observation from previous animal work [10,43] that suggests that endogenous levels of NO may serve to protect the lung from hyperoxic lung injury [43]. Based upon our current findings, we suspect that, once F_E_NO levels fall below a critical level, the antioxidant defense and other processes in the lung that depend on NO become overwhelmed by the hyperoxic stress, resulting in changes in lung function and symptoms of PO_2_tox. However, as discussed in a review paper by Lui et al. [44], the role of the various NOS isoforms in the generation of NO in the face of hyperoxic stress and the impact of NO in the pathogenesis of acute lung injury is still under debate.

Several studies have attempted to ascertain the underlying mechanisms responsible for the decrease in F_E_NO with HBO exposures [45,46,47]. The common thesis of these studies centers around the hypothesis that the decrease in F_E_NO with hyperoxic exposures is due to decreased enzymatic generation of NO due to oxidation of tetrahydrobiopterin (BH_4_), which is an essential cofactor required for NO production by NOS [48]. Fismen et al. [45] found that increased O_2_ concentrations reduced BH_4_ levels in human endothelial cells in a dose-dependent manner, without directly affecting the NOS enzyme. Similarly, Hesthammer et al. [46] reported that BH_4_ levels in human umbilical vein endothelial cells (HUVEC) decreased in a dose-dependent manner. Although the latter study found that HUVEC NO production was also decreased following a 40 kPa O_2_ exposure, a further decrease in HUVEC NO production was not observed when the oxygen exposure was increased to 60 kPa. In a follow up study by Hesthammer et al. [47], in which BH_4_ was measured in venous blood samples of subjects exposed to 100% oxygen for 90 min at atmospheric pressure, both F_E_NO and BH_4_ significantly decreased when measured 10 min after the exposure. Although oxidation of BH_4_ levels and its subsequent uncoupling/inhibitory effects of NOS on NO production appear to be a plausible reason for the reduced F_E_NO with hyperoxic exposures; other mechanisms include the reaction of oxygen or superoxide radicals with NO to form peroxynitrite, which likely also contribute to the reduced F_E_NO.

### Study Strengths and Limitations

To our knowledge, this is the first study that combined measurements of F_E_NO with traditional measures of pulmonary function in healthy divers to assess PO_2_tox susceptibility following provocative HBO exposures that resulted in significant decrements in lung function. While the current study involved a small number of subjects, the study design incorporated multiple baseline and recovery measurements of F_E_NO and pulmonary function to provide a robust indication of daily inter-individual variation and accurately define when these dependent variables fell significantly outside of the individual’s normal range, following the HBO exposure. The results clearly showed a wide individual variability in pulmonary function changes resulting from the HBO exposures, with half of our subject population showing minimal changes in lung function following the six-hour dive and the other half showing significant decreases that were more than two standard deviations below their normal day-to-day range. While this experimental design allowed us to analyze individual susceptibility to PO_2_tox, the small *n* approach leaves group statistical analysis susceptible to type II errors from the large variability in individual responses to the HBO stress. However, given our primary aim, we felt the small *n* approach was ethically more defensible as a pilot study on individual PO_2_tox susceptibility than a larger *n* study with limited individual pre-dive data but a higher power to detect group-level changes in pulmonary function post-dive.

An additional limitation of the current study is that only two subjects completed a control (normobaric air) condition, and that the study design was unblinded. This may have led to experimenter and subject bias regarding the expectation of pulmonary function decrements and PO_2_tox symptoms following the HBO exposures. While performing the pulmonary function measurements in accordance with the ATS recommendations [15,16,17,18] helps to reduce this potential bias, most spirometry measurements are dependent upon the individual performing a maximal inspiratory and/or expiratory effort to determine if pulmonary function is affected by the HBO exposure. In contrast, measurements of F_E_NO are conducted at a fixed expired flow rate and do not require maximum effort by the subject. F_E_NO may thus offer an alternative or complementary assessment of pulmonary hyperoxic stress that is less prone to the subject’s effort than traditional spirometry measurements. While we acknowledged that there are many sources of NO in the lungs that can contribute to F_E_NO, and that the underlying mechanistic role of NO in hyperoxic acute lung injury is still controversial, F_E_NO may provide a useful noninvasive marker of the hyperbaric oxidative stress response of the lungs and lead to new insights into individual susceptibility to PO_2_tox.

## Figures and Tables

**Table 1:** Mean baseline levels, 2 × coefficient of variation (CV), and percent change in F_E_NO (expired flow rate = 50 mL/s) following the 202.65 kPa HBO exposures. The subject data (rows) are ordered from highest to lowest baseline F_E_NO. The baseline CV was determined from 10 measurements taken over 5 consecutive days before the dive (see Methods). Post-dive 1 and post-dive 2 measurements were taken between 15 min and 1 h post dive. Recovery measurements (Rec 1, 2, 3) were taken 1-, 2-, and 3-days post-dive.

Subject	Baseline F_E_NO		Post-Dive 1 6 h O_2_	Post-Dive 2 8 h O_2_	Rec 1	Rec 2	Rec 3
	Mean ppb	CV × 2 (%)					
5	59	24%	−55%	−65%	−11%	0%	−6%
7	44	32%	−58%	−67%	+5%	0%	+22%
1	41	16%	−44%	−61%	−5.7%	+28%	−4%
8	38	30%	−46%	−60%	+14%	+76%	+36%
2	24	22%	−51%	NA	+37%	+3%	+15%
4	24	34%	−65%	NA	+9%	+40%	+10%
3	21	34%	−57%	NA	−5%	+2%	+4%
9	19	20%	−64%	NA	+55%	+34%	+66%
Mean	34 ppb	26.5%	−55% †	−63% †	+12%	+23%	+18%

† Group mean F_E_NO significantly different from baseline (*p* < 0.0001). NA = Not applicable. Cells highlighted in grey indicate the time points where significant decrements in pulmonary function were observed (see Table 2, Table 3 and Table 4).

**Table 2:** Mean baseline levels, 2 × coefficient of variation (CV), and percent change in FVC following the 202.65 kPa HBO exposures.

Subject	Baseline FVC		Post-Dive 1 6 h O_2_	Post-Dive 2 8 h O_2_	Rec 1	Rec 2	Rec 3
	Mean (L BTPS)	CV × 2 (%)					
5	5.61	4.9%	−0.8%	+1.9% *	−1.5%	−0.4%	−0.6%
7	4.22	6.3%	+1.9%	−0.5% *	+0.9%	−2.8%	−1.9%
1	5.99	2.2%	+4.3%	−2.8% *	−0.5%	−0.7%	−4.2%
8	4.91	6.8%	+4.7%	+3.3% *	+3.3%	−0.4%	−0.8%
2	5.58	7.0%	+3.6% *	NA	+2.9%	−1.6%	−1.3%
4	5.49	6.6%	−3.5% *	NA	−2.2%	−0.4%	−2.7%
3	4.78	5.3%	−3.1% *	NA	+1.3%	−5.2%	−0.8%
9	5.49	5.3%	−9.3% *	NA	−17.3% *	−7.7%	−5.4%
Mean	5.26 L	5.6%	−0.3%	+0.5%	−1.6%	−2.4%	−2.2%

* = Symptoms of PO_2_tox reported. NA = Not applicable. Cells highlighted in grey indicate significant decrements in FVC from the individual’s mean baseline.

**Table 3:** Mean baseline levels, 2 × coefficient of variation (CV), and percent change in F_I_VC following the 202.65 kPa HBO exposures.

Subject	Baseline F_I_VC		Post-Dive 1 6 h O_2_	Post-Dive 2 8 h O_2_	Rec 1	Rec 2	Rec 3
	Mean (L BTPS)	CV × 2 (%)					
5	6.15	5.5%	−0.7%	+0.5% *	−2.4%	−0.8%	−0.2%
7	4.57	4.4%	+2.0%	+3.9% *	−1.8%	−0.4%	+2.6%
1	6.93	3.3%	−2.6%	−0.7% *	−0.3%	+0.7%	−0.3%
8	5.35	3.2%	+4.9%	+4.9% *	+5.8%	+6.5%	+1.9%
2	6.43	4.4%	−6.8% *	NA	−4.0%	−1.4%	−1.7%
4	6.61	6.0%	−3.4% *	NA	+0.5%	−2.1%	+5.6%
3	5.43	2.7%	−18.8% *	NA	−5.0%	−0.9%	−5.0%
9	6.15	5.9%	−15.1% *	NA	−12.5% *	−12.5%	−10.4%
Mean	5.95 L	4.5%	−5.1%	+2.2%	−2.5%	−1.4%	−0.9%

* Symptoms of PO_2_tox reported. Cells highlighted in grey indicate significant decrements in F_I_VC from baseline. NA = Not applicable.

**Table 4:** Mean baseline levels, 2 × coefficient of variation (CV), and percent change in D_L_CO following the 202.65 kPa HBO exposures.

Subject	Baseline D_L_CO		Post-Dive 1 6 h O_2_	Post-Dive 2 8 h O_2_	Rec 1	Rec 2	Rec 3
	Mean (mL/mmHg/min)	CV × 2 (%)					
5	37.9	8.7%	+3.2%	+2.6% *	+0.3%	+1.3%	−5.0%
7	26.1	7.0%	−2.2%	−5.6% *	−2.2%	−5.6%	−2.2%
1	41.1	9.5%	−3.1%	−7.3% *	−17.0%	−6.5%	−6.8%
8	33.1	11.2%	−3.1%	−4.3% *	−10.1%	−9.8%	−0.4%
2	40.6	13.9%	−10.1% *	NA	−7.7%	−15.1%	−21.7%
4	45.5	15.4%	−17.8% *	NA	−20.0%	−4.0%	−10.7%
3	39.6	6.9%	−16.6% *	NA	+3.6%	−8.5%	−3.2%
9	38.3	8.7%	−13.0% *	NA	−9.6% *	−27.6%	−20.8%
Mean	37.8	10.2%	−7.8%	−3.7%	−7.8%	−9.5%	−8.9%

* Symptoms of PO_2_tox reported. Cells highlighted in grey indicate significant decrements in D_L_CO from baseline. NA = Not applicable.

## Data Availability

The data from the current study are not publicly available due to government restrictions regarding data sharing, but are available from the corresponding author on reasonable request and when requirements are met.

## Appendix A. Diffusion Capacity for Carbon Monoxide Measurements Corrected for Hb and V_A_ and Additional Spirometry Measurements Taken Pre- and Post-Dive

**Table A1:** Mean baseline levels, 2 × coefficient of variation (CV), and percent change in D_L_CO adjusted for hemoglobin (D_L_CO _adj_) following the 202.65 kPa HBO exposures.

Subject	Baseline D_L_CO _adj_		Post-Dive 1 6 h O_2_	Post-Dive 2 8 h O_2_	Rec 1	Rec 2	Rec 3
	Mean (mL/mmHg/min)	CV × 2 (%)					
5	38.6	9.4%	+6.5%	+1.3% *	+1.3%	+1.3%	−2.6%
7	25.5	6.9%	−1.6%	−5.9% *	−2.0%	−2.0%	−1.2%
1	39.9	9.9%	−3.5%	−6.0% *	−18.8%	−5.8%	−3.3%
8	31.9	11.8%	−1.5%	−6.2% *	−8.4%	−6.2%	+1.3%
2	39.3	13.4%	−8.0% *	NA	−6.0%	−13.9%	−22.1%
4	45.0	15.2%	−16.9% *	NA	−20.9%	−6.7%	−10.9%
3	38.9	8.3%	−16.9% *	NA	+3.2%	−8.9%	−4.5%
9	38.5	9.0%	−13.0% *	NA	−11.4% *	−30.4%	−21.1%
Mean	37.2	10.5%	−6.9%	−4.2%	−7.9%	−9.1%	−8.1%

* Symptoms of pulmonary O_2_ toxicity reported. Cells highlighted in grey indicate significant decrements in D_L_CO_adj_ from baseline. NA = Not applicable.

**Table A2:** Mean baseline levels, 2 × coefficient of variation (CV), and percent change in D_L_CO adjusted for alveolar volume (D_L_CO/V_A_) following the 202.65 kPa HBO exposures.

Subject	Baseline D_L_CO/V_A_		Post-Dive 1 6 h O_2_	Post-Dive 2 8 h O_2_	Rec 1	Rec 2	Rec 3
	Mean (mL/mmHg/min)	CV × 2 (%)					
5	4.82	7.2%	+6.8%	+3.5% *	−1.5%	+3.7%	−1.0%
7	4.37	8.9%	−2.3%	−11.0% *	−5.2%	−9.6%	−3.7%
1	4.97	8.5%	−3.8%	−7.2% *	−14.5%	−7.4%	−10.3%
8	5.10	7.1%	−8.0%	−6.9% *	−12.5%	−17.6%	−4.3%
2	5.29	11.3%	−2.3% *	NA	−9.6%	−14.7%	−13.2%
4	5.85	10.3%	−9.6% *	NA	−16.8%	−9.9%	−12.5%
3	6.10	5.8%	−1.1% *	NA	−4.1%	−12.8%	−2.1%
9	5.05	9.5%	+3.6% *	NA	+15.8% *	−15.6%	−11.5%
Mean	5.19	8.6%	−2.1%	−5.4%	−6.1%	−10.5%	−7.3%

* Symptoms of pulmonary O_2_ toxicity reported. Cells highlighted in grey indicate significant decrements in D_L_CO/V_A_ from baseline. NA = Not applicable.

**Table A3:** Mean baseline levels, 2 × coefficient of variation (CV), and percent change in alveolar volume (V_A_) following the 202.65 kPa HBO exposures.

Subject	Baseline V_A_		Post-Dive 1 6 h O_2_	Post-Dive 2 8 h O_2_	Rec 1	Rec 2	Rec 3
	Mean (L BTPS)	CV × 2 (%)					
5	7.86	4.1%	−0.3%	−0.9% *	+1.05	−2.3%	−3.8%
7	5.97	4.2%	0.0%	+5.7% *	+3.4%	+4.0%	+1.7%
1	8.28	8.6%	+0.4%	−0.2% *	−3.2%	+0.1%	+4.0%
8	6.50	5.1%	+5.4%	+2.8% *	+2.8%	+9.7%	+4.2%
2	7.68	7.9%	−8.2% *	NA	+2.7%	−0.3%	+0.3%
4	7.77	8.4%	−9.1% *	NA	−3.7%	+6.7%	+2.2%
3	6.49	5.8%	−15.9% *	NA	+8.0%	+4.9%	−1.1%
9	7.59	5.2%	−16.3% *	NA	−22.1% *	−14.4%	−10.8%
Mean	7.27	6.2%	−5.5%	+1.9%	−1.4%	+1.1%	−0.4%

* Symptoms of pulmonary O_2_ toxicity reported. Cells highlighted in grey indicate significant decrements in V_A_ from baseline. NA = Not applicable.

**Table A4:** Mean baseline levels, 2 × coefficient of variation (CV), and percent change in F_E_V1 following the 202.65 kPa HBO exposures.

Subject	Baseline F_E_V1		Post-Dive 1 6 h O_2_	Post-Dive 2 8 h O_2_	Rec 1	Rec 2	Rec 3
	Mean (L BTPS)	CV × 2 (%)					
5	3.97	5.3%	−0.8%	+6.3% *	−0.5%	+1.3%	−3.3%
7	3.08	6.1%	+0.6%	−2.3% *	−1.3%	−5.5%	−5.8%
1	3.96	3.9%	+2.8%	−2.5% *	−1.3%	+0.3%	−2.3%
8	3.81	6.4%	+2.1%	+5.5% *	+3.1%	−3.1%	−4.2%
2	4.28	7.2%	+3.3% *	NA	+0.9%	−1.6%	−1.9%
4	4.16	7.5%	−2.4% *	NA	−5.5%	−3.4%	−5.5%
3	3.30	7.9%	−8.5% *	NA	−4.5%	+0.3%	+0.3%
9	3.96	6.7%	−6.1% *	NA	−20.2% *	−9.1%	−6.3%
Mean	3.82	6.4%	−1.1%	+1.8%	−3.7%	−2.6%	−3.6%

* Symptoms of pulmonary O_2_ toxicity reported. Cells highlighted in grey indicate significant decrements in F_E_V1 from baseline. NA = Not applicable.
